# Bumblebees (*Bombus terrestris*) Improve ‘Hass’ Avocado (*Persea americana*) Pollination

**DOI:** 10.3390/plants10071372

**Published:** 2021-07-05

**Authors:** Raphael A. Stern, Ada Rozen, Ravit Eshed, Tali Zviran, Isaac Sisai, Amir Sherman, Vered Irihimovitch, Gal Sapir

**Affiliations:** 1 MIGAL—Galilee Research Institute, P.O. Box 831, Kiryat Shmona 11016, Israel; gal.sapir@edetepta.com; 2 Department of Biotechnology, Faculty of Life Sciences, Tel-Hai College, Upper Galilee 12210, Israel; 3 The Volcani Center, Institute of Plant Sciences, Agricultural Research Organization, Bet Dagan 50250, Israel; adar@volcani.agri.gov.il (A.R.); reshed@volcani.agri.gov.il (R.E.); taliz@volcani.agri.gov.il (T.Z.); sissai@volcani.agri.gov.il (I.S.); asherman@volcani.agri.gov.il (A.S.); veredi@volcani.agri.gov.il (V.I.)

**Keywords:** avocado (*Persea americana*), bumblebee (*Bombus terrestris*), honeybee (*Apis mellifera*), pollination, yield

## Abstract

Pollination is limiting for avocado production. We examined whether adding bumblebees (BBs; ca. 10 hives/ha) to conventional honeybees (HB; 5 hives/ha) would improve ‘Hass’ avocado pollination and yields. A preliminary trial (2017/18) in an avocado orchard with four consecutive rows of ‘Hass’ followed by one row of ‘Ettinger’ serving as a pollenizer (20% ‘Ettinger’) showed a considerable increase in ‘Hass’ yield in rows adjacent to (up to 80 m from) the BB hives vs. distant rows (=controls). In 2018/19, the trials were extended to three additional orchards. A significant yield increase was obtained in the BB hive-adjacent trees compared to BB hive-distant ones. Similar results were obtained in 2019/20, in experiments conducted throughout the country. The SNP analysis, to determine the parents of ‘Hass’ fruit at varying distances from the BB hives, showed no differences in the cross-pollination rate (‘Hass’ × ‘Ettinger’). However, pollination rates and the number of germinating pollen grains per stigma decreased with distance from the hives, and correlated to the negative gradient in yield. Taken together, our data suggest that adding BB hives to ‘Hass’ avocado orchards, at ca. 10 hives/ha resulting in 0.5–1.0 BB visits/tree per min, increases pollination and, accordingly, total yield.

## 1. Introduction

Insufficient pollination is a major limiting factor for avocado (*Persea americana* Mill.) fertility [1,2,3]. In Mediterranean regions, and in particular in Israel, the honeybee (*Apis mellifera*; HB) is the main commercial pollinator of avocados. In most avocado orchards, HB hives are introduced during flowering, but these are not sufficient for avocado pollination because they prefer citrus and wildflowers, such as mustard, over avocado flowers [4,5]. In addition, in commercial orchards, HBs are ineffective cross-pollinators at distances larger than one or two rows from the pollenizer, thus decreasing the yield of most cultivars that require cross-pollination for maximum yield [6]. Even after doubling their density from 2.5 to 5.0 hives/ha in some Israeli avocado orchards, we did not observe any yield improvement (unpublished data). Various reasons have been suggested to explain the relatively low attractiveness of avocado flowers to bees, including small-sized flowers, unsuitable nectar sugar composition and relatively high sucrose levels [7]. Furthermore, Afic et al. [8,9] reported that the low attractiveness of the flowers to the bees is due to the nectar composition, rather than the sugars within it; specifically, they showed that avocado nectar is relatively rich in a wide range of minerals, primarily potassium and phosphorus. These minerals have a significant rejection effect at the concentrations found in avocado nectar [8,9].

The bisexual flowers of avocado exhibit ‘synchronous protogynous dichogamy’. Avocado cultivars are classified as type A or B, based on their flowering behavior [10]. Each avocado flower opens twice over two consecutive days. In type A cultivars, the flowers open in the morning in the female stage (with a receptive stigma), close at midday and reopen in the afternoon of the following day, in the male stage. In type B cultivars, the flowers open in the afternoon of the first day in the female stage, close in the evening and reopen the following morning in the male stage. This strategy promotes outcrossing; however, self-fertilization can occur if the female and male phases overlap [11]. Currently, cv. Hass (type A) dominates the world avocado market [10]. ‘Hass’ is also the main avocado cultivar in Israel, and its preferred pollinator is ‘Ettinger’ (type B).

Studies have observed many visitors other than HBs on avocado flowers [12,13,14]. Due to the relatively low attractiveness of avocado blossoms to HBs, the effectiveness of alternative pollinators in Israel has been tested. As a first approach, the original pollinator of avocado flowers in native Mexico was sought (HBs are not the main pollinators there). A number of stingless bee species were identified as being potential commercial pollinators, including *Scaptotrigona mexicana*, Apidea and Meliponinae [15], and a number of their hives were brought to Israel for quarantine, but they did not survive Israel’s hot summer temperatures.

A second approach was to examine the possibility of using bumblebees (*Bombus terrestris* L.; BBs) to supplement the HBs. Indeed, Ish-Am et al. [6] reported a 66% increase in ‘Ettinger’ crops in plots where BB hives were added to HB hives. In ‘Hass’, on the other hand, the positive effect of BBs on the crop was minimal, and was obtained only in rows far from the ‘Ettinger’ pollenizer and not in rows adjacent to it, regardless of the location of the BB hives. Despite indications of crop improvement following the introduction of BBs to avocado orchards in Israel, they were never used commercially, due to inconsistent results and their minimal impact on ‘Hass’.

BBs have several attributes that are beneficial for pollination. Like HBs, BBs collect pollen from numerous sources [16], but their pollen-carrying capacity is greater [17], due to their body size, which is about twice as large [16]. In addition, on each foraging trip, BBs generally visit many more flowers than HBs [18,19,20]. Moreover, BBs forage at temperatures below 14 °C, the limiting temperature for HB activity [20,21], and under harsher conditions [22]. BB pollination can thus begin earlier in the day and is not halted by inclement weather. On the other hand, the BB hive holds many less bees: approximately 200, compared to approximately 30,000 in the HB hive [22,23].

Zhang et al. [24], studying peach pollination in a greenhouse, found that the addition of BBs to HBs improves the fruit set and fruit size, compared to pollination with HBs alone. They attributed this to an increase in the number of pollen grains landing on the stigmas, leading to faster germination rates of the pollen tube in the styles. Similar results were found in Asian pear (*Pyrus pyrifolia*) pollinated in excess [25]. Keeping this notion in mind, in the last few years we decided to test the efficacy of BBs as a supplement to HBs in Israel, in orchards of deciduous fruit trees such as pears and apples [19,20]. We found improved HB pollination behavior in apple orchards due to a positive interaction with BBs [20]. Moreover, the impressive increase in cross-pollination of that species resulted in an increase in the number of seeds in the fruit, which led to a significant improvement in the number of fruit per tree and fruit size. This approach has become commercial practice in apple orchards in recent years. In litchi, a fruit species that can self-fertilize like avocado but is very attractive to HBs [26], we found that the addition of BBs to the orchards results in an increase of about 50% in fruit set and yield [27]. The yield increase was obtained because of a significant improvement in cross-pollination, due to the higher pollination efficacy of BBs as pollen gatherers, unlike HBs, which mainly collect nectar [27].

Our working hypothesis was that the addition of BBs to ‘Hass’ avocado orchards, to supplement the standard stocking with HBs, will improve pollination and lead to higher yield.

## 2. Materials and Methods

The experiments were conducted during the years 2017–2020 in mature ‘Hass’ and ‘Ettinger’ avocado orchards located in Northern and Central Israel (Table 1), at about 100–200 m above sea level. All orchards included only ‘Hass’ as the main cultivar and ‘Ettinger’ as the pollenizer, in different ratios and arrangements [28] (see Table 1).

### 2.1. Hive Introduction

**Figure 1:**
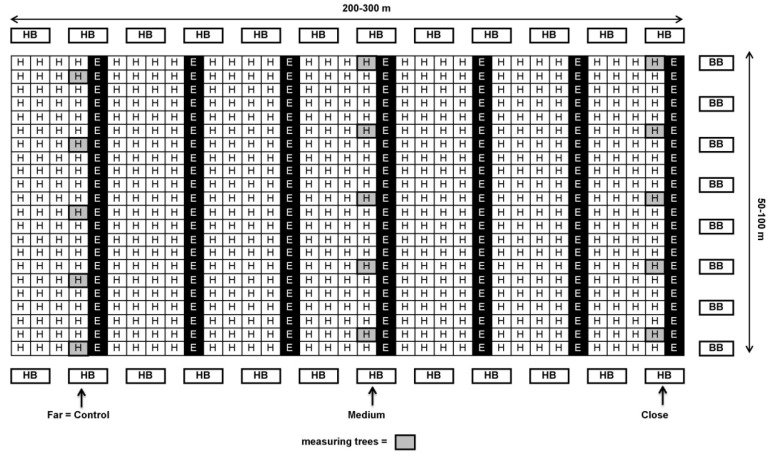
Schematic design of ‘Ettinger’ and ‘Hass’ plot at a 1:4 ratio (Kfar Giladi and Regba) with honeybee (HB) hives at the beginning and end of the rows and bumblebee (BB) hives on only one side of the orchard (right) or on both sides (not shown on the map). Close: 30–100 m; medium: 100–200 m; far: 200–300 m from BB hives.

**Figure 2:**
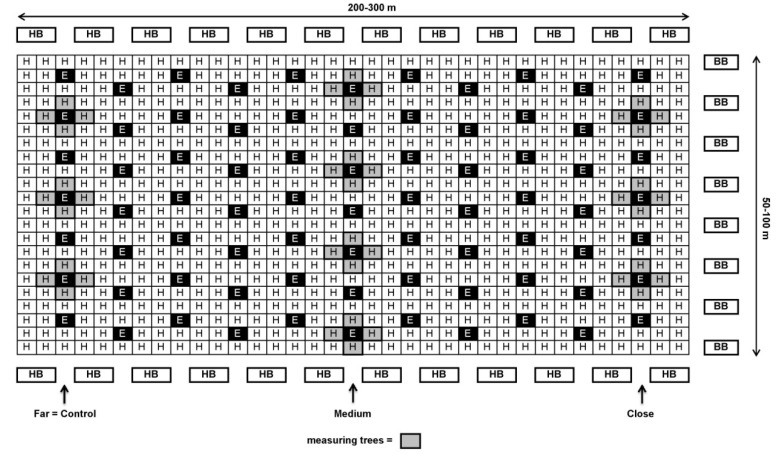
Schematic design of ‘Ettinger’ and ‘Hass’ plot at a 1:9 ratio (Kfar Hanassi and Eyal) with honeybee (HB) hives at the beginning and end of the rows and bumblebee (BB) hives on only one side of the orchard (right) or on both sides (not shown in the Figure). Close: 30–100 m; medium: 100–200 m; far: 200–300 m from BB hives.

About a week prior to the beginning of bloom during the first half of April, BB hives with 200 workers each (“BioBee”, Kibbutz Sde Eliyahu, Israel and “Pollination Services”, Kibbutz Yad Mordechai, Israel) were placed on one or both sides of each orchard at different ratios (5–12 hives/ha, Table 1). A week later, HB hives (30,000 workers each) were introduced at a rate of 5 hives/ha, along the top and bottom edges of the orchard (see Figure 1 and Figure 2). The reason for the earlier introduction of BB hives was that in contrast to HB it takes about 1 week until the BB start foraging.

### 2.2. Tree Marking to Measure Bee Activity, Yield, and Fruit Size

In each orchard, 4–10 ‘Hass’ trees with similar medium flowering intensity were selected for each distance from the BB hives. Each tree serves as one replicate. The trees selected in orchards with an ‘Ettinger’-to-‘Hass’ ratio of 1:4—Kfar Giladi and Regba, were from the ‘Hass’ row adjacent to the ‘Ettinger’ row (see Figure 1). In orchards with an ‘Ettinger’-to-‘Hass’ ratio of 1:9—Kfar Hanassi and Eyal, the selected trees were always around the ‘Ettinger’ pollenizer trees (Figure 2).

### 2.3. HB and BB Foraging Activity

HB and BB foraging activity (number/tree per min) on the selected trees (see Section 2.2) was assessed by an observer who counted the bees on whole trees using a hand-held counter while circling the tree at a distance of approximately 1.0 m for 60 s [5,29,30]. The number of HBs or BBs per tree was measured almost daily throughout the flowering season, usually from 10:00 to 12:00 h, when the bees show peak activity.

### 2.4. Yield and Fruit Weight

The number of fruit and yield per marked tree were recorded at harvest. Average fruit weight from each tree was determined from 100 randomized fruit per tree.

### 2.5. Pollination Rate and Number of Pollen Grains on the Stigma

Pollination rate was examined only in Kfar Giladi in April 2019. On 30 April, 100 ‘Hass’ flowers (20 flowers per tree × 5 trees) from each of the three distances (60, 120, and 180 m) from the BB hives were sampled randomly. The flowers were collected at the end of their female phase [7] and were fixed in FAA (70% ethanol:glacial acetic acid:formalin (18:1:1, *v*/*v*)) [31,32]. At the end of the flowering season, the flowers were washed three times with distilled water, 1 h per wash, and left overnight in 5% (*w*/*v*) sodium sulfite. On the following day, the flowers were autoclaved at 1 kg/cm^2^ for 10 min in 5% sodium sulfite to soften the tissues, and stained with 0.1% (*v*/*v*) aniline blue in 0.1% (*w*/*v*) K_3_PO_4_ for callose [33]. Preparations were observed under a Zeiss Axio Vert.A1 microscope with UV epifluorescence.

### 2.6. Molecular Paternity Analysis

We collected 180 average-size ‘Hass’ fruit during the harvesting seasons of 2018/19 and 2019/20 from BB hive-adjacent trees and BB hive-distant trees (15 fruit per tree × 3 trees × 2 distances, in each season). Fruit were collected from the experiments carried out in Kfar Giladi (2018/19) and Kfar Hanassi (2019/20), respectively. Following germination of the fruit seeds, DNA was extracted from leaves sampled from each seedling, and from ‘Hass’ and ‘Ettinger’ trees, essentially as described previously [34].

To identify the pollen donor (‘Hass’ or ‘Ettinger’), DNA samples were diluted 1:20 and genotyped using 24 informative single nucleotide polymorphisms (SNPs), selected from 109 SNPs used in an earlier study to characterize the Israeli Avocado Germplasm Bank (see [34] and Table S1). The analysis was applied to 90 and 89 germinated seedlings originating from fruit collected during 2019 and 2020, respectively. SNP genotyping was performed on a Fluidigm 192.24 Dynamic Array using the genotyping EP1 System (San Francisco, CA, USA). Fluorescence intensity was measured with the EP1 reader (Fluidigm) and plotted on two axes. Genotypic calls were made using the Fluidigm SNP Genotyping Analysis program.

### 2.7. Statistical Analysis

Data were analyzed for statistical significance among means using JMP software (SAS Institute Inc., Cary, NC, USA). Duncan’s new multiple range test was applied to compare treatments when ANOVA showed significant differences between the means at *p* ≤ 0.05.

## 3. Results

### 3.1. First Year—2017/18

**Figure 3:**
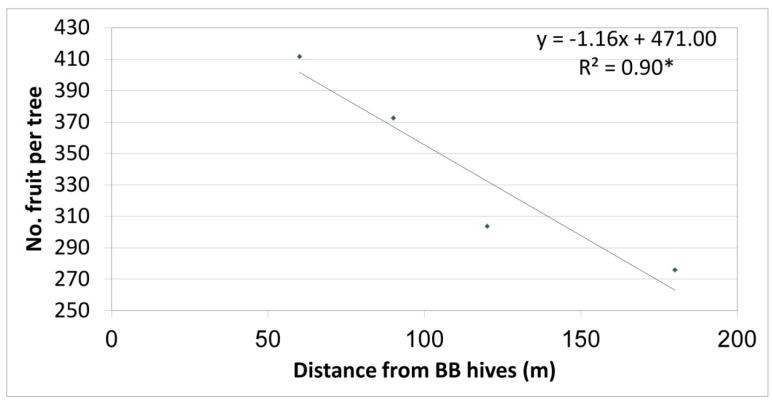
Relationship between row distance from bumblebee (BB) hives and the number of fruit per ‘Hass’ tree, Kfar Giladi, 2017/18. Each data point is an average of five trees, * *p* < 0.05.

Yield results of the first (preliminary) year depicted a significant negative correlation between the number of fruit per tree and BB hive distances from the row (Figure 3 shows four distances in this year). As such, an average of 410 fruit per tree was obtained in trees located about 60 m from the BB hives, and the number of fruit decreased linearly as the distance increased. An average of 270 fruit per tree was obtained in the farthest row, 180 m from the BB hives (control with no BBs on the trees).

### 3.2. Second Year—2018/19

#### 3.2.1. Bee Activity

The activities of HBs and BBs on the trees were first examined in Kfar Hanassi during April 2018 on two sides of the plot: close to the BB hives (10–50 m) and far from the BB hives (180–230 m). Throughout the sampling period, ‘Ettinger’ was more attractive than ‘Hass’ to both HBs and BBs at each distance (Table 2). However, for BBs, there was a difference in their numbers between the distances, for both ‘Ettinger,’ and ‘Hass’ (Table 2). No interaction between bees (HB and BB), distances (close and far), or cultivar (Ettinger and Hass) was found.

#### 3.2.2. Yields

In Kfar Hanassi, ‘Hass’ yields were recorded for the trees closest to the BB hives (20 m) to those farthest from the BB hives (230 m). We found a negative gradient of the number of fruit and yield with increasing distance from the BB hives (Table 3). The harvest closest to the BB hives (20 m away) had the largest number of fruit per tree; at the second distance (50 m), there was a considerable and significant reduction in fruit number (Table 3). From this distance outward, a farther, although not always significant reduction in fruit number was recorded (Table 3). Taken together, the addition of about 300 fruit/tree, with no significant impact on fruit weight, resulted in a crop increase of over 20 ton/ha.

In Kfar Giladi, the experiment was carried out in the same orchard as in the previous year. Results of this trial again showed that the number of fruit per tree was negatively correlated to distance from the BB hives (Table 4). Since the average fruit weight was almost the same for all three distances, the yield per tree and per ha was correlated to the number of fruit per tree.

Although the yield of the far distance was high (55 kg/tree) the yield of the trees close to the BB hives increased significantly, by another 21 kg/tree.

The experimental design in Regba was almost identical to that in Kfar Giladi (Table 1) and the yield data were similar: a higher number of fruit and yield per tree for the rows up to approximately 70 m from the BB hives, with no significant influence on fruit weight (Table 5).

### 3.3. Third Year—2019/20

#### 3.3.1. Bee Activity

In view of the possible correlation between the increased activity of BBs on ‘Hass’ trees in the rows closest to the hives (April 2018) and the high yield obtained in those trees (February 2019), we expanded the bee observations to additional orchards in the next year. Field observations were conducted for several consecutive days in Kfar Giladi, with daily monitoring carried out for 11 days of flowering. The observations were made on five trees having a similar (medium) flowering intensity for each distance × three distances (60, 120, and 180 m from BB hives = close, medium, and far) × two cultivars.

In a comparison of cultivars, bee activity on the ‘Ettinger’ trees was again significantly higher than on the ‘Hass’ trees: HBs on the ‘Ettinger’ trees averaged 28.6 bees/tree per min over the entire period compared to 6.9 for the ‘Hass’ trees (no daily data shown). A similar ratio was found for the BBs: 0.1 BBs/tree per min on ‘Ettinger’ vs. 0.02 for the ‘Hass’ trees. Both types of bees prefer Ettinger, probably due to higher secretion of nectar in flowers and due to higher sugar concentration in nectar (A. Dag—personal communication).

**Figure 4:**
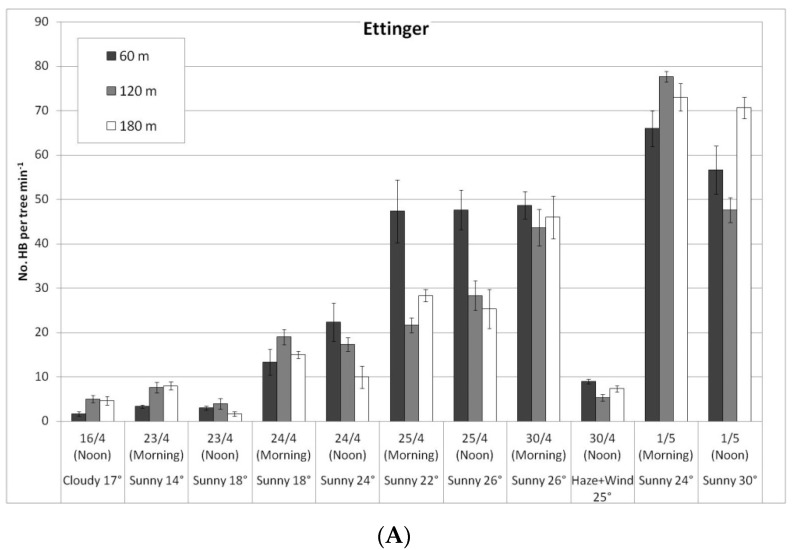

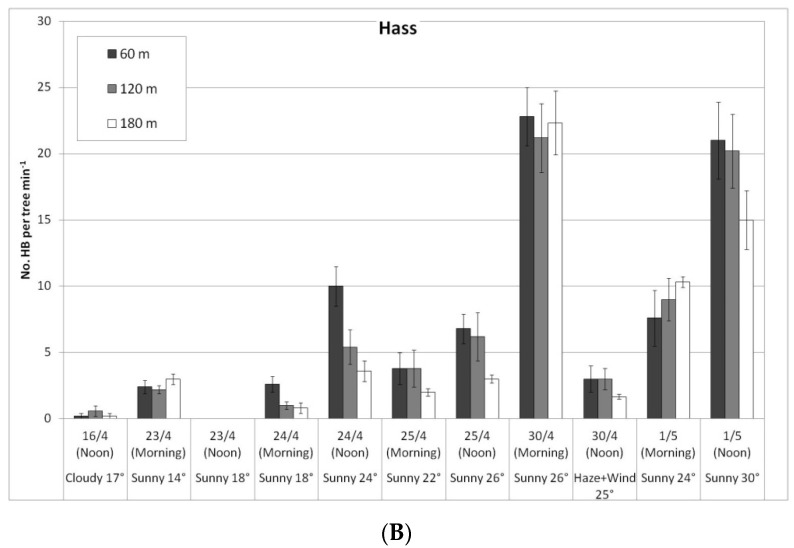
Daily number of honeybees (HBs) on ‘Ettinger’ (**A**) and ‘Hass’ (**B**) trees at three distances from the bumblebee (BB) hives (60, 120, and 180 m), Kfar Giladi, April 2019. Data are means of five trees at each distance ± SE.

**Figure 5:**
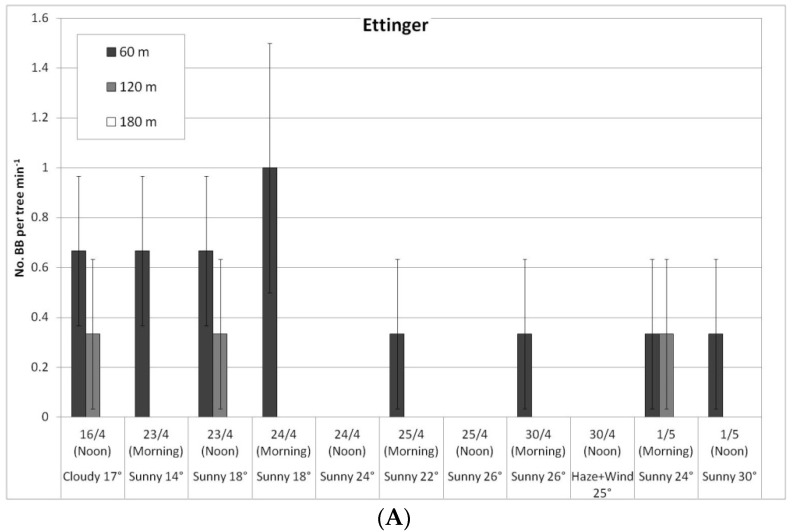

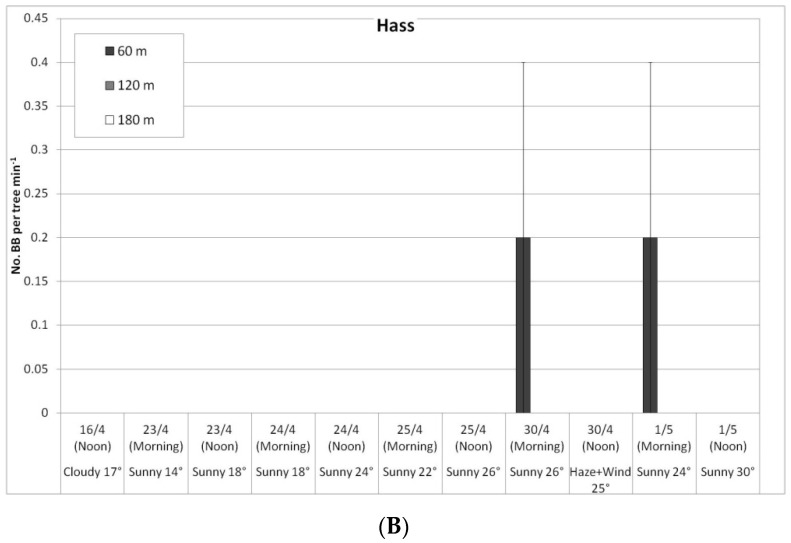
Daily number of bumblebees (BBs) on ‘Ettinger’ (**A**) and ‘Hass’ (**B**) trees at three distances from the BB hives (60, 120, and 180 m), Kfar Giladi, April 2019. Data are means of five trees at each distance ± SE.

In a comparison of the distances, daily HB activity on the trees was similar for all distances, on both ‘Ettinger’ (Figure 4A) and ‘Hass’ (Figure 4B). However, BB activity differed with distance. The BBs, which were located on only one side of the orchard, were almost always seen only a short distance from the BB hives, and sometimes (only for ‘Ettinger’), even at the medium distance. No BBs were observed at all at the far distance (Figure 5A,B).

**Figure 6:**
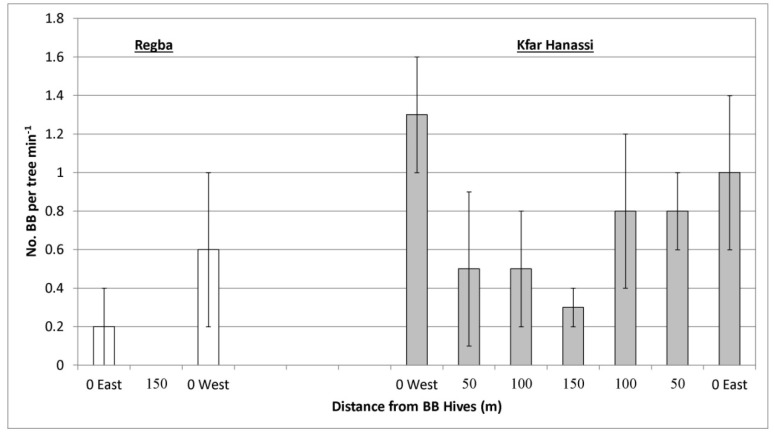
Number of bumblebees (BBs) on ‘Hass’ trees at three distances (Kfar Hanassi, 2 May 2019) or seven distances (Regba, 5 May 2019) from the BB hives. Data are the means of five ‘Hass’ trees at each distance ± SE.

A single follow-up of bee activity was conducted at full bloom in two other experimental plots, Regba (2 May 2019) and Kfar Hanassi (5 May 2019). In both, BB hives were placed on both sides of the plot (Table 1) to examine the bee-activity gradient, yield gradient, and maximum distance of BB efficiency. Observations were conducted on five ‘Hass’ trees at each of the three different distances in Regba, and on four trees at each of seven distances in Kfar Hanassi. Like Kfar Giladi, there were no differences in HB activity on the ‘Hass’ trees at the different distances (data not shown), whereas BB activity changed with distance in both orchards. Considerable BB activity was observed only close to the hives in both Regba and Kfar Hanassi, although the differences in activity at the various distances were insignificant (Figure 6).

#### 3.3.2. Yield

The fruit in Kfar Giladi were harvested at 60, 90, and 120 m from the BB hives. A considerable difference in the number of fruit and yield per tree was found between the nearest and farthest distances—300 fruit/tree (43 kg/tree) at 60 m compared to 105 and 125 fruit/tree (16 and 19 kg/tree) at 90 and 120 m, respectively. This is a three-fold increase with increasing proximity of the BB hives.

**Figure 7:**
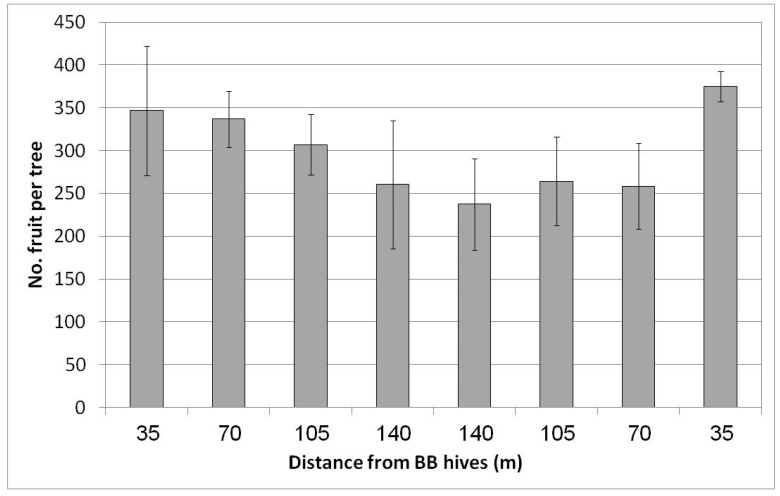
Effect of adding bumblebee (BB) hives (April 2019) to both sides of the orchard on the number of fruit per tree in February 2020 at all distances from the BB hives, Regba, 2019/20. Data are the means of five ‘Hass’ trees at each distance ± SE.

In Regba (April 2019), two groups of BB hives were placed east and west of the plot. A negative yield gradient was obtained from the nearest distance to the BB hives (35 m west and east of the plot) to the largest distance at the center of the plot, approximately 150 m to the hives on each side (Figure 7). The gradual yield decline toward the plot center was not statistically significant but was nevertheless considerable. At the largest distance at the center of the plot, we counted about 250 fruit/tree (50 kg/tree) compared to the rows adjacent to the hives, where we counted about 360 fruit/tree (68 kg/tree). Hence the BBs contributed about 100 fruit to the tree, equivalent to 5 ton/ha. No statistical difference in fruit size was found for the different distances from the BB hives (data not shown).

**Figure 8:**
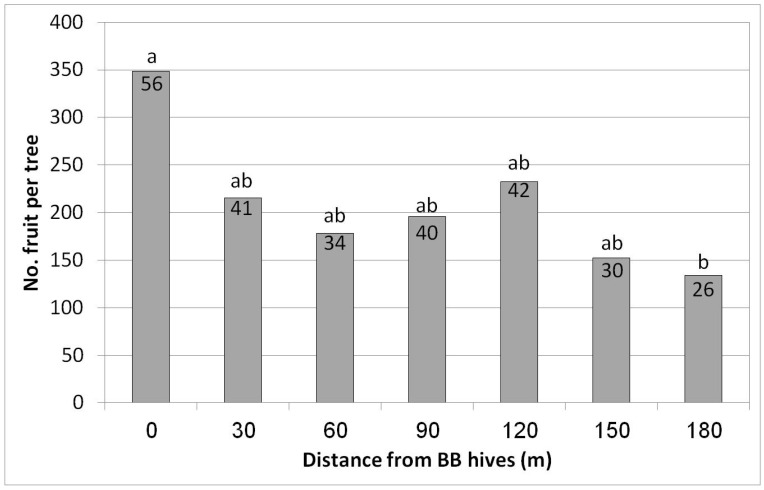
Effect of adding bumblebee (BB) hives (April 2019) on the number of fruit per tree and kilogram of fruit per tree (no. in the column) in February 2020 at seven distances from the BB hives, Eyal, 2019/20. Results in a column followed by different letters differ significantly according to Duncan’s new multiple range test, *p* < 0.05. Data are the means of five ‘Hass’ trees at each distance.

In Eyal, the highest yield was obtained in the first row closest to the hives (348 fruit/tree = 56 kg/tree, equivalent to 20 ton/ha) (Figure 8). However, a significant decrease was already obtained for the second distance, i.e., only 30 m from the hives (215 fruit/tree = 41 kg/tree). Lower yields were obtained for all of the other distances, with the lowest yield (26 kg/tree) at the largest distance, 180 m from the hives—about half of the yield from the closest hive row (56 kg/tree). No statistical difference in fruit size was found among the different distances from the BB hives (data not shown).

**Figure 9:**
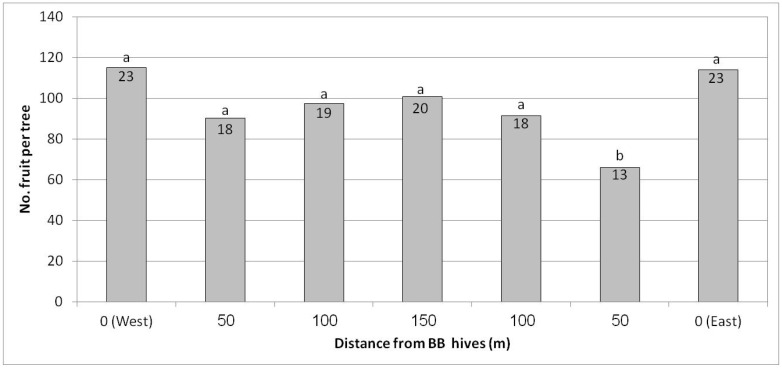
Effect of adding bumblebee (BB) hives (April 2019) to both sides of the orchard on the number of fruit per tree and kilogram of fruit per tree (no. in the column) in February 2020 at seven distances from the BB hives, Kfar Hanassi, 2019/20. Results in a column followed by different letters differ significantly according to Duncan’s new multiple range test, *p* < 0.05. Data are the means of 120 ‘Hass’ trees per distance (30 trees/row × 4 rows).

In Kfar Hanassi, we tested a commercial harvest for the first time, in contrast to the other trials where we had monitored fruit number and yield in randomly selected trees. In Kfar Hanassi, each row was harvested separately, and the average yield per tree was calculated. Each of the seven distances measured comprised four rows, representing four replicates per distance. Results, shown in Figure 9, indicated that despite the season’s low yield, the number of fruit and overall yield per tree were high at both ends of the plot near the BB hives compared to the center, about 120 fruit/tree vs. 70–100 fruit/tree, respectively. The average yield increase was 30%, from 90 to 120 fruit/tree. There was no statistical difference in fruit size between the different distances from the BB hives (data not shown).

#### 3.3.3. Pollination Rate

To determine whether the contribution of BBs to the increase in fruit per tree was caused by larger pollination and fertilization rates, which ultimately resulted in better fertilization, fruit set, and yield, in the spring of 2019, we conducted a pollination test in Kfar Giladi on flowers of the same trees on which we monitored bee activity and yield. In the near distance, 94% of the sampled flowers had pollen grains on the stigmas, whereas at the larger distances from the hives, pollination rates dropped (Table 6). Another interesting finding was that the number of pollen grains on the stigmas decreased significantly as the distance from the hives increased. As the distance from the hive increased, both pollination rates of the flowers and number of pollen grains per stigma decreased, hindering the chances of fertilization. The correlation between pollination rates and the number of grains per stigma was high and significant (*R*^2^ = 0.96).

#### 3.3.4. Molecular Paternity Analysis

The above data supported the notion that the addition of BB hives to ‘Hass’ avocado orchards increases pollination and, accordingly, total yield. To determine whether the contribution of BBs to the increment in the number of fruit per tree was also reflected in an increase in cross-pollination rates, we utilized SNP genotyping to define the pollen donor of ‘Hass’ fruit that were sampled from BB hive-adjacent trees (up to 50–60 m from the hives), and from BB hive-distant trees (up to 230–240 m from the hives). DNA extracted from leaves of the seedlings grown from seeds of the collected ‘Hass’ fruit was subjected to analysis (see Materials and Methods). Due to suspected technical problems in the Fluidigm reactions (no call results), only 20 of the 24 SNP markers were found suitable for the paternity analysis. Using the information obtained from these SNPs, we determined that 95.5–100% of the ‘Hass’ fruit sampled at different distances from the BB hives, during the two consecutive seasons, were the outcome of cross-pollination (‘Hass’ × ‘Ettinger’) (Table 7). In contrast, only 0–4.5% of the fruit collected from either BB hive-adjacent trees or BB hive-distant trees resulted from ‘Hass’ self-fertilization. The results suggested that the introduction of BB hives to the avocado orchards did not affect the cross-pollination rates (‘Hass’ × ‘Ettinger’), which were a priori very high.

## 4. Discussion

Our working hypothesis was that pollen limitation in avocado orchards does not allow for full crop potential, as observed by Pattemore et al. [35], and by Reilly et al. [36] for some other fruit tree species. Preliminary experiments in which we doubled the density of HBs from 2.5 to 5.0 hives/ha for avocado, as we had done with apple and pear [29,30], did not improve the yield (unpublished data). To overcome this limitation, we tested whether the addition of BBs, which are known to improve pollination in general, and cross-pollination in particular [6], would have an impact on ‘Hass’ avocado pollination rates and yields. Results of our study, which lasted three consecutive years and was carried out in various orchards, showed that pollen limitation indeed exists and that the introduction of BBs, as an additional pollinator, significantly increased the number of fruit per tree. However, treatment efficiency was restricted to short distances from the BB hives; as the distance from the hives increased, the treatment efficiency decreased. These results are in line with Marques et al. [37] who showed that improved almond fertility, following the addition of BB hives in Spain, was only obtained at distances close to the BB hives; at farther distances, the yield increase was marginal [37]. Knowing that in order to fly, BBs need to generate heat so as to increase the temperature of their thoracic muscles to 30 °C, and that a single trip is energetically very costly for them [22], it is reasonable to assume that the energetic cost of the flight might be the reason why BBs prefer to visit ‘Hass’ flowers close to their hives (thus spending as little energy as possible).

The additional crop yield achieved in the near distances (close to the hives) usually ranged from 50 to 100%. Furthermore, despite a very high yield in the trees furthest from the BB hives (a type of “control”), considerable and significant improvement was achieved closer to the BB hives, ranging more or less between 10 and 20 ton/ha and about 20 and 40 ton/ha.

As a result of the increase in the number of fruit per tree, there was sometimes a slight and insignificant reduction in the weight of individual fruit, which did not reduce the overall crop. However, it should be noted that an excessive crop load of >500 avocado fruit per tree may result in a drastic reduction in fruit size along with significant damage to flowering and harvest in the following year [38].

Field observations of the activity of HBs and BBs on the trees showed no difference in the number of HBs per tree throughout the orchard, but BBs were seen only at distances close to their hives—up to 80 m in Regba and Kfar Giladi, and 35 m in Kfar Hanassi. Similar results have been reported by Wolf and Moritz [39]. In our study, the effectiveness of BBs as pollinators was reflected in the pollination rates of ‘Hass’ flowers, which decreased with increasing distance of the trees from the hives. Moreover, a positive and significant correlation between the above pollination rates and the number of pollen grains per stigma was found and therefore, not only did the chances of flower pollination increase, but so did the potential for fertilization [40]. The optimal number of pollen grains needed on avocado stigmas to complete the fertilization process is unclear, ranging from 20 grains in some reports [7,41] to only 1–5 grains [1]. Most researchers claim that this is one of the major causes of a low avocado fruit set, although the correlation is not always clear. Indeed, Alcaraz and Hormaza [1] found a positive correlation between the number of pollen grains on the stigma and fertilization ratios. A similar result was first reported by Shoval [41], who also noticed the “population effect”, that is, the more grains on the avocado stigma, the greater the chances for increased germination and fertilization. However, factors additional to pollination can affect the odds of the fruit set, such as flower quality and perfection, starch concentration in the pistil, temperature during flowering, and more [42,43,44].

The decrease in efficiency of BBs to shorter distances in Kfar Hanassi (35 m) compared to Regba and Kfar Giladi (80 m) is likely due to the arrangement and percentage of ‘Ettinger’ trees as foreign pollenizers in the plot. In Kfar Giladi and Regba, where there were four consecutive rows of ‘Hass’ followed by a row of ‘Ettinger’ (20% ‘Ettinger’), pollination efficiency was achieved at an ultimate distance of 70–80 m from the hives. In Kfar Hanassi and Eyal, where fewer ‘Ettinger’ trees were planted (11% ‘Ettinger’), the BBs were more effective closer to the hive, at 20–50 m. It seems that the shorter distance efficiency in Kfar Hanassi is unrelated to hive number since we had the same effect in the 2 years of experimentation with a different number of hives placed in the same area (Table 1, Kfar Hanassi 2018/19 vs. 2019/20). In light of this, it is necessary to calibrate the optimal number of hives (i.e., find the optimal density) for each pollenizer/plot structure in the orchards to determine the optimal distance between each hive and plot.

Although the number of BBs was very low compared to the number of HBs, in both Regba in 2019 and Kfar Hanassi in 2019, with no BB activity, the tree crop was relatively low, and with as few as 0.5 BBs/tree per min, the crop significantly increased. As a result, we assume that if more BBs were present in the orchard (i.e., 1 BB/tree per min), an additional increase in yield would occur.

As previously mentioned, the introduction of BB hives to the orchard might lead to an increase in yield due to an increase in overall cross-pollination rates. Previous studies performed in Israel, aimed at defining cross-pollination rates of ‘Hass’ using isozymes as molecular markers, revealed that in orchards containing ‘Ettinger’ as the pollinator, 87–90% of the ‘Hass’ progeny in trees adjacent to the pollenizer were the outcome of fertilization by ‘Ettinger’ [45,46,47]. Similar results were obtained in a recent study comparing ‘Iriet’ and ‘Ettinger’ as pollenizers of ‘Hass’, using SNPs for paternal identification, which showed that mature ‘Hass’ self-fertilization rates were only 3.1% and 0% in two consecutive seasons [28]. In line with the former report, here, our paternity analysis indicated that regardless of distance from the BB hives, the vast majority of the ‘Hass’ fruit (95.5–100%) were the outcome of cross-pollination with ‘Ettinger’, suggesting that in this case, yield enhancement was mainly due to improvement of the pollination process.

It should be noted, however, that it is still unclear whether the improvement in pollination is due to the additional pollinator itself—the BB, which is less affected by the low attractiveness of avocado flowers and transfers more pollen grains between the male phase of the pollenizer and the female phase of ‘Hass’ flowers [35], or to a positive interaction of BBs with HBs, which increases HB mobility between cultivars. In fact, we found support for the second hypothesis in apple [20] and pear [19]. Enhancement of HB mobility after BB visits can be explained by the fact that HBs dislike variable resource distribution, especially when they contain empty flowers [48]. Earlier BB visits can deplete flower rewards (pollen and nectar) from a large number of flowers. HBs that generally prefer to forage flowers close to each other will change their behavior and move more to the side [49]. This change in behavior might cause a better pollen flow between the pollenizers and the main cultivar [50,51].

## 5. Conclusions

In summary, we showed the contribution of BBs to improving pollination and increasing yield of the ‘Hass’ avocado. However, the exact mechanism responsible for pollination enhancement in the avocado still needs to be determined. The exact mechanism warrants further study, along with determinations of optimal distances and densities per unit area of BB hives.

## Figures and Tables

**Table 1:** Avocado plots used for bumblebee (BB) pollination experiments during the years 2017–2020.

Year	Orchard	Location	Orchard Design and Tree Data					BB Design	
			Tree Spacing (Rows × Trees) (m)	Trees/ha (No.)	Ettinger: Hass Ratio	Age (Years)	Height (m)	BB Density (Hives/ha)	BB Hive Location (One or Two Sides of the Plot) ^3^
2017/18	Kfar Giladi	Upper Galilee	6 × 4	420	1:4 ^1^	6	4	8	1
2018/19	Kfar Giladi	Upper Galilee	6 × 4	420	1:4 ^1^	7	5	8	1
	Kfar Hanassi	Hula Valley	6 × 4	420	1:9 ^2^	15	6	8	1
	Regba	Western Galilee	7 × 4	350	1:4 ^1^	10	6	10	1
2019/20	Kfar Giladi	Upper Galilee	6 × 4	420	1:4 ^1^	8	6	8	1
	Kfar Hanassi	Hula Valley	6 × 4	420	1:9 ^2^	16	6	12	2
	Regba	Western Galilee	7 × 4	350	1:4 ^1^	11	6	12	2
	Eyal	Central Israel	7 × 4	350	1:9 ^2^	6	4	5	1

^1^ 1:4 = Four consecutive ‘Hass’ rows followed by ‘Ettinger’ as the fifth row (20% ‘Ettinger’, see Figure 1). ^2^ 1:9 = Each third row of ‘Hass’ has one ‘Ettinger’ tree for every two ‘Hass’ trees (11% ‘Ettinger’, see Figure 2). ^3^ See Figure 1 and Figure 2.

**Table 2:** Number of honeybees (HBs) and bumblebees (BBs) per tree per min on ‘Ettinger’ vs. ‘Hass’ at Kfar Hanassi, April 2018.

Pollinator	Cultivar	Distance from BB Hives		Total Average
		Close	Far	
HB	Ettinger	8.90 Aa	11.00 Aa	10.00 a
	Hass	1.50 Ab	1.90 Ab	1.70 b
BB	Ettinger	0.44 Aa	0.10 Ba	0.27 a
	Hass	0.07 Ab	0.02 Bb	0.05 b

Results in a column for each pollinator separately (HB or BB) followed by different lowercase letters and results in a row for each pollinator separately (HB or BB) and each cultivar separately (Ettinger or Hass) followed by different uppercase letters that differ significantly according to Duncan’s new multiple range test, *p* < 0.05. Data are the means of four ‘Ettinger’ trees and eight ‘Hass’ trees × two distances (close = 0–50 m and far = 180–230 m from the BB hives) × 5 days (April 9, 12, 15, 16, and 17).

**Table 3:** Effect of adding bumblebee (BB) hives (April 2018) on the number of fruit per tree, fruit weight, and total yield (February 2019) at six distances from the BB hives, Kfar Hanassi, 2018/19.

Distance from BB Hives (m)	No. Fruit/Tree	Fruit Weight (g)	Yield	
			kg/Tree	ton/ha ^1^
20	496 a	219 a	108 a	45 a
50	328 b	232 a	72 b	30 b
80	222 bc	219 a	48 bc	20 bc
140	173 c	217 a	38 c	16 c
170	202 bc	219 a	43 bc	18 bc
230	232 bc	208 a	50 bc	21 bc

Results in a column followed by different letters differ significantly according to Duncan’s new multiple range test, *p* < 0.05. Data are the means of eight ‘Hass’ trees at each distance. ^1^ Equivalent to ton/ha.

**Table 4:** Effect of adding bumblebee (BB) hives (April 2018) on the number of fruit per tree, fruit weight and total yield (February 2019) at three distances from the BB hives, Kfar Giladi, 2018/19.

Distance from BB Hives		No. Fruit/Tree	Fruit Weight (g)	Yield	
Name	m			kg/Tree	ton/ha ^1^
Close	30–70	492 a	158 a	76 a	32 a
Medium	100–200	406 ab	169 a	67 ab	28 ab
Far	230–270	335 b	167 a	55 b	23 b

Results in a column followed by different letters differ significantly according to Duncan’s new multiple range test, *p* < 0.05. Data are the means of 10 ‘Hass’ trees at each distance. ^1^ Equivalent to ton/ha.

**Table 5:** Effect of adding bumblebee (BB) hives (April 2018) on the number of fruit per tree, fruit weight, and total yield (February 2019) at three distances from the BB hives, Regba, 2018/19.

Distance from BB Hives		No. Fruit/Tree	Fruit Weight (g)	Yield	
Name	m			kg/Tree	ton/ha ^1^
Close	30–70	445 a	195 a	85 a	30 a
Medium	100–230	338 b	209 a	68 b	24 b
Far	260–320	320 b	207 a	65 b	23 b

Results in a column followed by different letters differ significantly according to Duncan’s new multiple range test, *p* < 0.05. Data are the means of 10 ‘Hass’ trees at each distance. ^1^ Equivalent to ton/ha.

**Table 6:** Pollination rate and number of pollen grains per stigma of ‘Hass’ flowers at Kfar Giladi, 30 April 2019.

Distance from Bumblebee Hives (m)	Pollination Rate (%)	Grains per Stigma (No.)
60	94	4.5
120	76	2.5
180	67	2.1
Significance	NS	*

Data are the means of 100 flowers (20 flowers per tree × 5 trees) for each distance. * *p* < 0.05, NS = not significant.

**Table 7:** Paternity analysis of ‘Hass’ fruit sampled from trees at different distances from the bumblebee (BB) hives.

Season/Orchard	Seedling Number	Distance from BB Hives (m)	Pollen Donor Identity	
			‘Hass’	‘Ettinger’
2018/19 Kfar Giladi	90 (100%)	60	0 (0%)	45 (100%)
		240	2 (4.5%)	43 (95.5%)
2019/20 Kfar Hanassi	89 (100%)	50	1 (2.2%)	44 (97.8%)
		230	0 (0%)	44 (100%)

## Data Availability

All data, tables and figures in this manuscript are original.

## Supplementary Materials

The following are available online at https://www.mdpi.com/article/10.3390/plants10071372/s1, Table S1: List of the primers used in this study. For each of the 24 SNPs, we specified the amplicon sequences as well as its two alleles and the contig where the SNP is located (see Rubinstein et al., 2020). For each SNP—Specific Target Amplification primers (STAs), Locus Specific primers (LSPs) and Allele Specific primers (ASPs) are provided.
