# Supplementary material for: Bumblebees (Bombus terrestris) Improve ‘Hass’ Avocado (Persea americana) Pollination
Source: Plants (Basel). 2021 Jul 5;10(7):1372. doi: 10.3390/plants10071372 (PMC8309347; doi:10.3390/plants10071372)
Supplement: Supplementary file 1 [file plants-10-01372-s001.zip › plants-1266186-supplementary.pdf]

Table S1. List of the primers used in this study. For each of the 24 SNPs, we specified the amplicon sequences as well as its two alleles and the contig where the SNP is located (see Rubinstein et al., 2020). For each SNP - Specific Target Amplification primers (STAs), Locus Specific primers (LSPs) and Allele Specific primers (ASPs) are provided.

	SNP Name	Contig	Position	Allele1	Allele2	STRAND	STA_primer	LSP_primer	ASP1_primer	ASP2_primer	AMPLICON_SEQ
**1**	AV 003 301_10	g30150t00001	810	C	T	forward	CATTCAAAACAACCAGTGAATAGATTAG	TCTCAGACTGCTTTGGCTTCTCC	TGGGGCACAAATAAGGGATGCC	TGGGGCACAAATAAGGGATGCT	CATTCAAAACAACCAGTGAATAGATTAGGAGCTGGGCGAAGAGGGTGGGGCACAAATAAGGGATGC[T/C]AGGGATGGAGGAGTCAGTGAGGGAGAAGCCAAAGCAGTCTGAGA
**2**	AV 005 099_40	g09915t00001	540	A	G	forward	CCAGTCAAAATGCCCTGAAGAAA	TGACTCTGATAGTTGGAGTGATTCTTG	CTAAGATCTACCATGATACTTCCGACATA	CTAAGATCTACCATGATACTTCCGACATG	CCAGTCAAAATGCCCTGAAGAAACTCGAGCAGCAGGATGGCTAAGATCTACCATGATACTTCCGACAT[G/A]CTTTAACAATGGCAGTTCAAGAATCACTCCAACTATCAGAGTCA
**3**	AV 011 221_46	g22101t00001	1346	C	G	reverse	AATGATATGGCTTGTTAAAACGCTT	GTCCTGAATCCGATCGAAGTAAGTAC	TCTTGTCAAGGTCTTAGCCTCCTG	TCTTGTCAAGGTCTTAGCCTCCTC	AATGATATGGCTTGTTAAAACGCTTCTTGTCAAGGTCTTAGCCTCCT[G/C]TTTCCATGTGAGTACTTACTTCGATCGGATTCAGGAC
**4**	AV 012 267_37	g26796t00001	137	C	T	reverse	GCATGGCCACTCTAGCCCA	AGGTCCACTTTGAGCCCCTATTTAT	CGGAGAGGCCTTTCCATAACCG	CGGAGAGGCCTTTCCATAACCA	GCATGGCCACTCTAGCCCACGGAGAGGCCTTTCCATAACC[A/G]AGACTGATAAATAGGGGCTCAAAGTGGACCT
**5**	AV 024 157_50	g15753t00001	550	C	G	reverse	GAAATCCCCAAAACTCATCAAACTAA	GTGAAGAGAAGGAAGATAACTGGAGAG	CCCTCTCTTTGCCTTTCAACTCG	CCCTCTCTTTGCCTTTCAACTCC	GAAATCCCCAAAACTCATCAAACTAACCCTTTTTATGAATTCTTCCCCTCTCTTTGCCTTTCAACTC[G/C]TCTTTCTATCTATTAGATGAAGATCTCTCCAGTTATCTTCCTTCTCTTCAC
**6**	AV 038 262_46	g26200t00001	546	A	T	reverse	TGTACTCACTCACCTTCTTCTTTTC	AGATCCCATGGCACGTTGATCG	CCACCACCTTCGTCGTGCCT	CCACCACCTTCGTCGTGCCA	TGTACTCACTCACCTTCTTCTTTTCCAATTTTGGGCCCAATGCCACCACCTTCGTCGTGCC[T/A]GCTGAGATCTTCCCTGCGAGGCTGCGATCAACGTGCCATGGGATCT
**7**	AV 054 048_97	g04881t00001	897	A	C	reverse	CCAAGCTCACTAATAAGCACGC	TTGACATAATTGGAGGGAATGCTCG	GAAAGATGCCAAAGTACTTCACGCT	GAAGATGCCAAAGTACTTCACGCG	CCAAGCTCACTAATAAGCACGCGTACTTCAGAAAGATGCCAAAGTACTTCACGC[T/G]CACGAGCATTCCCTCCAATTATGTCAA
**8**	AV 057 210_87	g21033t00001	1087	A	G	reverse	GTCATGACTACTAGCCTCAACTTAG	CATACTTGATGGGTGCCTTGAACC	AAAACTGATATGCCTATGAAGCCTTCT	AAACTGATATGCCTATGAAGCCTTCC	GTCATGACTACTAGCCTCAACTTAGCTAAGGCTGTGCAGAAAACTGATATGCCTATGAAGCCTTC[C/T]ATGCCCGTTTCAACCAGTCAGGTTCAAGGCACCCATCAAGTATG
**9**	AV 058 213_34	g21382t00001	834	C	T	forward	GCAATCATGGTGCAAATAACACAA	GTGGGAGATGAGGTATAGGATCGC	GCCCCTCGCAGCCTCCAAC	GCCCCTCGCAGCCTCCAAT	GCAATCATGGTGCAAATAACACAAGCCCCTCGCAGCCTCCAA[T/C]GCGATCCTATACCTCATCTCCCAC
**10**	AV 078 076_17	g07659t00002	517	A	T	reverse	ATTAAATACCCTGTTCAGGTTCAGAA	CGCCCACATCAGGAAAAGATTCATA	AAATGGAAAGGTTCTTTCACTGCCT	AAATGGAAAGGTTCTTTCACTGCCA	ATTAAATACCCTGTTCAGGTTCAGAAAAATGGAAAGGTTCTTTCACTGCC[A/T]GGTTATGAATCTTTTCCTGATGTGGGCG
**11**	AV 081 109_35	g10924t00001	235	A	C	forward	CATGGAGTAGGAATAGTAGTTTTAAAAAC	TCCTACATATGTATGTATGAGGGAGGG	AAAGATATGGCCATTAACGGGTTGA	AAAGATATGGCCATTAACGGGTTGC	CATGGAGTAGGAATAGTAGTTTTAAAAACTAGATAATCAAAGTTGACAAAGATATGGCCATTAACGGGTTG[C/A]TACAAATCCTCAACCCCCCTCCCTCATACATACATATGTAGGA
**12**	AV 087 055_34	g05557t00001					GCTCCTTCCTGTGCTCACTTT	TCAGAATGTTCACAATTCAGGCGG	GAAGAGTTCTAGGGATTAAGCTATTGTTA	GAAGAGTTCTAGGGATTAAGCTATTGTTC	GCTCCTTCCTGTGCTCACTTTCGAAGTAGTCAAGAAGAAGAGTTCTAGGGATTAAGCTATTGTT[C/A]GTGGGGTGTAATTCTGCCCGCCTGAATTGTGAACATTCTGA
**13**	AV 088 277_95	g27701t00001					CTCCTTCCCTACACCTATTACACA	GGGGTTCAATCATCAAACTTCACATG	AAAATCCATACATGATGGCTGAGTACA	AAATCCATACATGATGGCTGAGTACG	CTCCTTCCCTACACCTATTACACACTACCATCTAGATCGTATAAAAATCCATACATGATGGCTGAGTAC[A/G]TTAAGGACGTCAAAGCATGTGAAGTTTGATGATTGAACCCC
**14**	AV 101 088_32	g08815t00002					ATCCCTTGATATCAATTCATTGGAGA	CTGCGCGATATTCTGGCTAAAAAAG	GTAAGCCCCGGCATCAAACCT	GTAAGCCCCGGCATCAAACCG	ATCCCTTGATATCAATTCATTGGAGAATGTAAGCCCCGGCATCAAACC[T/G]CGCTTTTTTAGCCAGAATATCGCGCAG

**15**	AV 103131_12	g13103t00001	TCATCATAACCCAATGACACACTG	AATGGTCCCCACTTTGAGTCTGG	TCATCGTCGTCGTTGTTAGACACA	TCATCGTCGTCGTTGTTAGACACT	TCATCATAACCCAATGACACACTGCCATGCGTCCCCTGATCATCATCGTCGTCGTTGTTAGACAC[A/T]GTGGATGAAACATCAATGGCATCCCCAGACTCAAAGTGGGGACCATT
**16**	AV 107214_11	g21486t00001	TCTGGGAAACAGGTTTTCTGAGA	TCGATGTCATCCATGATTCGTAGAAC	ATGTCCTTGAAAGAAGCAGCTGAAG	ATGTCCTTGAAAGAAGCAGCTGAAT	TCTGGGAAACAGGTTTTCTGAGAAGGAATGGCGTGAGGTTTTTATGTCCTTGAAAGAAGCAGCTGAA[T/G]TGACGGTTCCAGGTTTTTTGAAGGTTCTACGAATCATGGATGACATCGA
**17**	AV 108249_68	g24900t00001	TGTTTGTACCATGTGACCCCAA	GAGATTTTCTGGCGCTGATGGTG	TCCTCAGATTTTAAAAATGACAACAGCTA	TCCTCAGATTTTAAAAATGACAACAGCTC	TGTTTGTACCATGTGACCCCAAAATAATAGTACAGACAGTCCTCAGATTTTAAAAATGACAACAGCT[C/A]CAATGGGTCCAGTCAAGACACCATCAGCGCCAGAAAATCTC
**18**	AV 114003_23	g00342t00001	TGGGAAAACCCTAAACAAGTTCG	GATCTCCGGCGTTCACTGAGC	GCAGCGCACATCTTGCAGGT	GCAGCGCACATCTTGCAGGA	TGGGAAAACCCTAAACAAGTTCGCAGCGCACATCTTGCAGG[A/T]CGCGGCGATCACCGGCTCAGTGAACGCCGGAGATC
**19**	AV 121031_57	g03109t00001	AGACTGTAGAAACTGAGACTGGC	TGAGGGTTTCCTCAGGTTACGTTT	CCGGAAGAATCCTATTTTAACTGATTCA	CCGGAAGAATCCTATTTTAACTGATTCT	AGACTGTAGAAACTGAGACTGGCCGGTCGAAGCGTAGCGTCCGGAAGAATCCTATTTTAACTGATTC[T/A]GGGTCATTTGAAAAGCCCAAACGTAACCTGAGGAAACCCTCA
**20**	AV 146072_75	g07246t00001	GGATAGCATCACAATCTCATCAAAAA	TGGGCTTCATTTAGACTTCCTGTTTT	AACTTTGTTAGAATAAGCGAAATATACAGTAC	AACTTTGTTAGAATAAGCGAAATATACAGTAT	GGATAGCATCACAATCTCATCAAAAACTTTTTAAAAGCAACTTTGTTAGAATAAGCGAAATATACAGTA[C/T]AAGAGATGTATATTTGAAAAACAGGAAGTCTAAATGAAGCCCA
**21**	AV 166149_06	g14937t00001	TGACAAAGCTTTCTTATTTATATATGTGAA	AGTCTATATAGGTTGTATGAAGTCAGGAC	ATGATCAAAGACCTAACAACCTTCTGT	ATGATCAAAGACCTAACAACCTTCTGA	TGACAAAGCTTTCTTATTTATATATGTGAAAAAAAGAACTCAATGAGAATGATCAAAGACCTAACAACCTTCTG[A/T]GCCAAAACTGGTCCTGACTTCATACAACCTATATAGACT
**22**	AV 167054_69	g05485t00002	CAAAAGCAAACTTTTATTGAGTGGC	TTCCTCTGTAGAAGTAGGCGGGT	AGTAACAAAACAAAAATATTCTTCATGCCG	AGTAACAAAACAAAAATATTCTTCATGCCT	CAAAAGCAAACTTTTATTGAGTGGCTAACAGTACAAGTAACAAAACAAAAATATTCTTCATGCC[G/T]ATCTATAGCAGAGGGCTCACGCCACCCGCCTACTTCTACAGAGGAA
**23**	AV 169254_87	g25456t00001	GAGAAGACTACAAGAAGTTGAGAGG	AATTGAAGGTACGTCCAAACAGGC	AAGTTCTGAAGGTATTTCTTATGTGTGT	AAGTTCTGAAGGTATTTCTTATGTGTGC	GAGAAGACTACAAGAAGTTGAGAGGTCTTTGCTAACAAGTTCTGAAGGTATTTCTTATGTGTG[C/T]GGTTGTCATTCTTCAGGGCCTGTTTGGACGTACCTTCAATT
**24**	AV 188021_32	g02123t00003	CTCTTTGAATAATTCTTCCCTCAACC	GTGCAAAATTCCTACCTACCAATTGG	TCTTGGAATTTGGATCCAGATAACTTTA	TCTTGGAATTTGGATCCAGATAACTTTC	CTCTTTGAATAATTCTTCCCTCAACCAGAAATCTTAGAATAGCTCTTGGAATTTGGATCCAGATAACTTT[A/C]TCAAGTATACCATGACGTGCTGGCCAATTGGTAGGTAGGAATTTTGCAC
